# Retraction Note: Development of grey wolf optimization based modified fast terminal sliding mode controller for three phase interleaved boost converter fed PV system

**DOI:** 10.1038/s41598-025-01790-w

**Published:** 2025-05-19

**Authors:** K. Krishnaram, T. Suresh Padmanabhan, Faisal Alsaif, S. Senthilkumar

**Affiliations:** 1 Department of EEE, E.G.S. Pillay Engineering College, Nagapattinam, Tamil Nadu India; 2 Department of Electrical Engineering, College of Engineering, King Saud University, 11421 Riyadh, Saudi Arabia; 3 Department of ECE, E.G.S. Pillay Engineering College, Nagapattinam, Tamil Nadu India

Retraction to: *Scientific Reports* 10.1038/s41598-024-59900-z, published online 22 April 2024

The Editors have retracted this Article.

Fig. 13 appears to be very similar to Fig. 9 in a previously-published article by different authors^1^. The Authors stated that they used the same laboratory; the laboratory, however, did not confirm this. The Editors no longer have confidence in the veracity of the data presented in this Article.

S. Senthilkumar disagrees with the retraction. K. Krishnaram, T. Suresh Padmanabhan, and Faisal Alsaif did not respond to correspondence from the Editors regarding this retraction.
